# Level of Adiponectin, Leptin and Selected Matrix Metalloproteinases in Female Overweight Patients with Primary Gonarthrosis

**DOI:** 10.3390/jcm10061263

**Published:** 2021-03-18

**Authors:** Jaromir Jarecki, Teresa Małecka-Massalska, Izabela Polkowska, Bartosz Potoczniak, Ewa Kosior-Jarecka, Imre Szerb, Ewa Tomaszewska, Martina Gutbier, Maciej Dobrzyński, Tomasz Blicharski

**Affiliations:** 1 Department of Rehabilitation and Orthopaedics, Medical University of Lublin, 20-059 Lublin, Poland; blicharski@vp.pl; 2 Department of Orthopaedics and Traumatology, Regional Hospital in Chełm, 22-100 Chełm, Poland; b.potoczniak@gmail.com; 3 Physiology Department, Medical University of Lublin, 20-059 Lublin, Poland; tmalecka@gmail.com; 4 Department and Clinic of Animal Surgery, University of Life Sciences, 20-033 Lublin, Poland; iza-polkowska@tlen.pl; 5 Department of Diagnostics and Microsurgery of Glaucoma, Medical University of Lublin, 20-079 Lublin, Poland; ekosior@poczta.onet.pl; 6 Department of Traumatology, Semmelweis University Budapest, 1085 Budapest, Hungary; szerbimre@gmail.com; 7 Department of Orthopaedics, Uzsoki Hospital Budapest, 1145 Budapest, Hungary; 8 Animal Physiology, University of Life Sciences, Lublin, 20-033 Lublin, Poland; ewa.tomaszewska@up.lublin.pl; 9 Department of Pediatric Dentistry and Preclinical Dentistry, Wroclaw Medical University, Krakowska 26, 50-425 Wrocław, Poland; martina.gutbier@umed.wroc.pl (M.G.); maciej.dobrzynski@umed.wroc.pl (M.D.)

**Keywords:** gonarthritis, obesity, female, pathogenesis, matrix metalloproteinases, adipokine, leptin

## Abstract

The aim of this study was to assess levels of adiponectin (ACRP-30) and leptin in serum and synovial fluid (SF) of overweight females with primary gonarthrosis (GOA) and to look for their correlations with clinical status and the level of biochemical OA biomarkers: matrix metalloproteinase (MMP) 1, MMP-9 and tissue inhibitor of metalloproteinase (TIMP-1). The studied group consisted of 39 overweight females undergoing primary total knee arthroplasty as a result of GOA. The stage of GOA was evaluated according to Knee Society Score (KSS), Ahlbäck and Kellgren–Lawrence (K-L) scores. Peripheral blood and SF were obtained. Levels of selected biomarkers were assessed using ELISA kits. The mean level of ACRP-30 in serum valued were 8393.80 ng/mL and in SF, 774.33 ng/mL, the mean concentration of leptin in serum was 32,040.74 pg/mL and in SF, 27,332.12 pg/mL. Levels of leptin in serum and SF correlated with body mass index (BMI), (*p* = 0.0005, and *p* = 0.0002, respectively). Levels of ACRP-30 in serum was correlated with clinical scores (Ahlbäck: *p* = 0.0214; K-L: *p* = 0.0146). ACRP-30 in SF correlated with ACRP-30 in serum (*p* = 0.0003), tended to negatively correlate with MMP-1 in serum (*p* = 0.0598) and positively correlate with pro-MMP-1 in SF (*p* = 0.0600). To conclude, this study confirms the correlations between concentrations of both, leptin and ACRP-30, comparing serum and SF. In overweight females, leptin levels increase with BMI and ACRP-30 serum level increase in more advanced GOA stages. Finally, leptin levels were correlated with TIMP-1 serum concentration, one of the biochemical markers of GOA.

## 1. Introduction

Osteoarthritis (OA) of the knee is a progressive disorder of the knee joints caused by gradual loss of cartilage and resulting in the development of bony spurs and cysts at the margins of the joints. OA is the most common form of arthritis and one of the leading causes of disability worldwide affecting around 250 million people [1,2]. The researches proved that OA is not exclusively a degenerative disease of the cartilage, but rather is a multifactorial entity with many possible causative factors such as trauma, mechanical forces, biochemical reactions, inflammation or metabolic derangements [3]. Moreover, OA is thought to be a disease of the entire joint involving cartilage, synovial membrane, meniscus and infrapatellar fat pad (IPFP) [4]. The OA incidence is predisposed to female gender with the ratio of about 1.7:1 [5], females also tend to have a more severe OA progression [6]. The increased OA frequency in postmenopausal women led to the hypothesis that female hormonal factors may play a causative role [7].

Obesity and being overweight have been recognized as pivotal risk factors for OA, especially gonarthrosis (GOA). The results from the Framingham Study demonstrated that women who had lost weight had a significantly reduced risk of developing symptomatic and radiographic GOA [8]. The Arthritis, Diet, and Activity Promotion Trial showed that combined weight loss and physical exercises were effective in decreasing pain and improving function in obese elderly patients with symptomatic GOA [9]. The overload observed in obesity gives an easy explanation for OA pathogenesis. However, metabolic factors were also pointed out in OA development affecting non-weight-bearing joints in overweight people [10]. Obesity is connected with low-grade inflammation and the loss of cartilage may be caused by an imbalance between pro- and anti-inflammatory cytokines resulting in catabolic effects.

Adipose tissue is related to OA development and progression by biomechanical, metabolic and proinflammatory factors triggering GOA. It has emerged as a potent internal endocrine organ, based on its ability to secrete biologically active adipokines, such as leptin and adiponectin, which are involved in different physiological processes. Leptin is primarily produced by adipose tissue. Circulating leptin levels correlate with the total amount of body fat and are increased in obese patients. On the contrary, adiponectin expression in adipose tissue and serum adiponectin levels are decreased in obese patients [11].

Proteolytic enzymes such as matrix metalloproteinases (MMPs) and aggrecanases are directly responsible for cartilage matrix degradation [12]. MMPs such as MMP-1, MMP-3, MMP-9 and MMP-13 play crucial roles in cartilage matrix degradation during OA [13]. Li et al. showed that MMP-3 and MMP-9 concentrations in plasma increased in patients with knee OA, and their levels correlated with the severity of clinical symptoms [14,15].

The aim of this study was to assess adiponectin (ACRP-30) and leptin levels in serum and synovial fluid (SF) of overweight females with primary GOA and to look for their correlations with clinical status and the level of biochemical OA biomarkers (MMP-1, MMP-9 and TIMP-1).

## 2. Methods

### 2.1. Studied Group

The studied group consisted of 39 post-menopausal females over 50 years of age with body mass index (BMI) over 25 undergoing primary total knee arthroplasty (TKA) because of knee osteoarthritis at the Department of Orthopaedics and Traumatology in Regional Hospital in Chełm, Poland, in 2019. Every patient, who fulfilled the inclusion criteria, did not meet any of the exclusion criteria and who signed a consent agreement form, was included to the study group. The study was conducted according to the Declaration of Helsinki and the design was accepted by Local Ethical Committee at the Medical University of Lublin, Poland (KE-0254/39/2019). BMI was calculated the day before surgery according to the formula: BMI = weight (kg): height (m)^2^.

The day before the surgery all patients had supine X-rays performed of both knees in anterior-posterior and lateral views. After evaluation of the X-rays in the studied group 28 patients had valgus knee deformation, 4—varus ones and 7 had both compartments affected. Afterwards, the stage of GOA was assessed according to the clinical Knee Society Score (KSS) (presented at www.orthopaedicscores.com, accessed on 7 January 2019) and when the results of X-rays were ready, modified radiological scales according to Ahlbäck [16] and Kellgren–Lawrence [17]. The Knee Society Score, updated and expanded in 2011, measures both objective and subjective outcomes.

The Ahlbäck scores divides GOA into 5 grades:

- grade 1: joint space narrowing (less than 3 mm)
- grade 2: joint space obliteration
- grade 3: minor bone attrition (0–5 mm)
- grade 4: moderate bone attrition (5–10 mm)
- grade 5: severe bone attrition (more than 10 mm).

The Kellgren and Lawrence system is another common method of classifying the severity of GOA using five grades. Its original description is as follows:

- grade 0 (none): definite absence of X-ray changes of osteoarthritis
- grade 1 (doubtful): doubtful joint space narrowing and possible osteophytic lipping
- grade 2 (minimal): definite osteophytes and possible joint space narrowing
- grade 3 (moderate): moderate multiple osteophytes, definite narrowing of joint space and some sclerosis and possible deformity of bone ends
- grade 4 (severe): large osteophytes, marked narrowing of joint space, severe sclerosis and definite deformity of bone ends.

All assessments were performed independently by two orthopedic surgeons (J.J. and B.P.) and mean values were used for further analysis. The mean values during clinical assessment did not differ for more than 10%.

The inclusion criteria were as follows:

1. Signed written consent for participations;
2. Female gender;
3. BMI over 25;
4. Age over 50 at diagnosis;
5. Fulfilling the criteria of the diagnosis of GOA according to the American College of Rheumatology:
  - Knee pain plus at least 1 of the following:
    (a) Age > 50 years,
    (b) Stiffness < 30 min,
    (c) Crepitus—plus osteophytes,
6. None of the exclusion criteria.

The exclusion criteria were as follows:

- Male gender
- Age below 50
- Previous surgical history on the knee prepared to TKA
- Previous injections to the knee prepared to the surgery with steroids or hyaluronans
- History of knee trauma (meniscal injury—removal of the medial or lateral meniscus by mini-arthrotomy, total rupture of anterior crucial ligament treated medically, trans-articular fracture of proximal tibial epiphysis)
- Ongoing knee infections or an infection within 3 months before planned surgery
- Ongoing or previous history of deep vein phlebitis
- Active dental or periodontal diseases
- Intake of any symptomatic slow acting drugs in osteoarthritis (glucosamine sulfate, chondroitin sulfate, collagen, Piascledine (ANGELINI, France)).

### 2.2. Obtaining and Evaluation of the Material

All blood samples were obtained from peripheral blood early in the morning on the day of admission to hospital from the patients with empty stomachs, then centrifuged, serum was portioned, frozen and stored at −40 °C for further tests. During TKA, after the incision of the knee joint capsule, the specimen of SF was obtained from the suprapatellar recess, which was then portioned, frozen and stored at −40 °C for further tests. In case of SF contamination with blood, the patient was excluded from the study. SF and serum were defrosted on the day of testing, one portion for a single test and evaluation of MMP-1, MMP-9 and TIMP-1 concentrations were performed using commercial ELISA kits (human Pro-MMP-1 Quantikine ELISA Kit (R&D System DMP100)(R&D Systems, Inc. Minneapolis, USA), human MMP-9 Quantikine ELISA Kit (R&D System DMP900), human TIMP-1 Quantikine ELISA Kit (R&D System DTM100). To obtain the concentration of adiponectin and leptin, following ELISA kits were used: human Leptin Quantikine ELISA Kit (R&D System DLP00), human total Adiponectin/Acrp30 Quantikine ELISA Kit (R&D System DRP300)). At the beginning, all tested samples of serum and synovial fluid were diluted 10 times with assay diluent according to the manufacturers’ instructions. All tests were conducted according to manufacturers’ instructions at the Chair and Department of Human Physiology, Medical University of Lublin, Poland. All quantitation was performed in duplicate, the mean value of 2 measurements was put as a result. The authors decided to reject the result if the difference between the single measurements was higher than 10% of higher value. Although, there were no such cases in all performed analyses.

### 2.3. Statistical Analysis

The statistical analysis of the results was performed with Statistica 13 software (www.statsoft.pl) We assessed the size of the sample retrospectively. We made the calculation based on the primary endpoint as continuous (adiponectin and leptin concentrations). Due to the post-hoc retrospective method, statistically significant results were analyzed. The post-hoc parameters also included anticipated means and we defined the types of errors (type I and type II errors). In the majority of studies, the *p*-value was determined to be below 0.05 in order to reject the null hypothesis, an error of type I (alpha) of 0.05 was used. In the case of type II (beta) error, the beta cut-off value in the majority of medical literature is established at 20% (0.2)—indicating a 20% chance of skipping a significant difference, therefore the type II error has been set at 0.2. Considering the correlation between primary endpoints in different materials analyzed in the study group (rho = 0.541 or rho = 0.892 for adiponectin and leptin, respectively), it was calculated that the minimum number of samples taken from the patients, which enables credible confirmation of the test hypothesis, is 24 or 7 samples, respectively. We considered values *p* < 0.05 as statistically significant. Moreover, we considered results *p* > 0.05 and *p* < 0.1 as having a tendency toward significance. The normality of data distribution was checked with the Kolmogorov–Smirnov test. In cases of all studied markers, non-normal distribution was observed. The results were reported mainly as mean ± SD (normally distributed data) or median and interquartile range (non-normally distributed data) or percentage values. In case of constant variables, Pearson’s test has been used to assess correlation between normally distributed data and Spearman’s rank correlation test (adjusted for statistically significant results) has been used in case of non-normally distributed data. The strength of the correlation was assessed on the basis of the following criteria: |r| = 0—no correlation; 0.0 < |r| ≤ 0.1—very weak correlation; 0.1 < |r| ≤ 0.3—weak correlation; 0.3 < |r| ≤ 0.5—medium correlation; 0.5 < |r| ≤ 0.7—strong correlation; 0.7 < |r| ≤ 0.9—very strong correlation; 0.9 < |r| < 1.0—almost complete correlation; and |r| = 1—full correlation. We considered results >0.05 and lower than 0.1 as having a tendency toward significance. All analyses regarding leptin results are adjusted according to BMI, as the leptin level may be influenced by BMI.

All results of correlations between studied markers and clinical variables were adjusted (with use of multiple regression) by age, BMI, KSS (clinical and functional), Alhbӓck score and Kellgren–Lawrence score.

## 3. Results

The studied group consisted of 39 females. The demographic and clinical characteristics are presented in Table 1. The mean values of studied biomarkers in blood serum and synovial fluid are presented in Table 2. Box plots representing data distribution of studied markers (measurements in serum and synovial fluid) was presented in Supplementary Figure S1.

The correlations between the studied markers were also examined. A strong correlation was observed between ACRP-30 level in serum and SF, and very strong correlation between both leptin concentrations. Moreover, leptin concentration in serum was correlated medium-strong to TIMP-1 concentration also assessed in serum. There was a tendency to negative correlation between leptin concentration in SF and the results in Kellgren–Lawrence scale.

We observed the correlations between the level of MMP-9 and pro MMP-1, when both were assessed in serum (R = 0.411, *p* = 0.0085) and SF (R = 0.380, *p* = 0.0155). Additionally, levels of ACRP-30 in SF had a tendency towards weak negative correlation with pro-MMP-1 in serum and tendency towards positive correlation to pro-MMP-1 level in SF.

When the results were adjusted for the remaining clinical variables (Age, BMI, KSS, Ahlbӓck score, Kellgren–Lawrence score) we found a moderate, positive correlation between serum ACRP-30 measured in serum and Ahlbӓck score (R = 0.393, *p* = 0.0214). However, the same marker after adjustment correlated moderately negatively with the results on the Kellgren–Lawrence score (R = −0.415, *p* = 0.0146). Moreover, leptin measured both in serum and SF correlated moderately positively with BMI (R = 0.568, *p* = 0.0005; R = 0.590, *p* = 0.0002, respectively). After adjustment for other clinical variables, MMP-9 measured in serum correlated moderately positively with BMI (R = 0.487, *p* = 0.0035). However, the same marker after adjustment correlated moderately negatively with the results of KSS functional (R = −0.361, *p* = 0.0360). After adjustment for other clinical variables, we found a tendency toward a statistically significant correlation between pro-MMP-1 measured in serum and BMI. Additionally, after adjustment, MMP-9 measured in serum shows a tendency toward significance in correlation with KSS (clinical). The detailed data on correlations between studied biomarkers and clinical variables are presented in Table 3. The details of all correlations between studied biomarkers are presented in Table 4.

## 4. Discussion

Knee joint infrapatellar fat pad (IPFP) located in close proximity to the synovium and cartilage have been found to produce adipokines and mediators contributing to OA pathogenesis [18,19,20] and seems to act as an anatomo-functional unit with the synovial membrane contributing to OA onset and progression [4,21]. Adipokines are considered potential risk factors for OA due to their associations with obesity [22]. When obesity is present, adipokines are significantly dysregulated and promote metabolic disorders [23]. The expression of adiponectin decreases with the increase of obesity, but some studies have shown the contrary results [24]. However, in this study analyzing overweight females, adiponectin levels in serum and SF were not significantly related to BMI. There are data suggesting that adiponectin secretion may differ according to adiposity levels and its distribution. In case of visceral adipose tissue, adiponectin levels decrease with the increase in adiposity, while in subcutaneous adipose tissue, the secretion of adiponectin remains unchanged, especially in women [25]. In this study the type of adipose tissue was not specified.

There are hypotheses that there might be differences in adiponectin levels based on the severity of OA, which could make it useful as a marker for disease severity [26,27]. In our study concerning obese female patients with advanced stages of GOA, the level of adiponectin in SF was 10 times lower compared to serum, which was also observed by other researchers [28,29]. It shows, that in obesity, the increase of adiponectin level in SF is not as pronounced as in serum, which might be connected with the fact that the main source of SF adiponectin in the knee joint is the IPFP, which does not grow with to the same extent compared to adipose tissue in the course of obesity. Additionally, it may also be related to the theory that the diminished SF adiponectin levels result from OA pathogenesis as a cause or a result of GOA. Our results are also compliant with the reports by Dong who observed diminished adipokine levels in OA patients with metabolic syndrome and suggested that these different expression patterns of adipokines may be partially caused by mechanical overweight stress on the joint [19].

The extent of OA may influence adipokine levels, as associations between adipokines and severity of the disease have been documented [26,27]. However, data on the association of plasma or SF adiponectin levels with OA severity are conflicting. In our results, exclusively concerning OA patients with advanced stages, higher concentrations of adiponectin in SF and serum were related to more advanced disease stages. Some studies have shown that adiponectin levels increased in erosive OA, probably mediating matrix degradation [26]. However, another study demonstrated diminished adiponectin levels in both plasma and SF as the severity of OA increased [30]. Finally, some studies [19] were unable to find a significant association between plasma or SF adiponectin levels and severity of OA. The above discrepancies in the results could be due to methodological issues regarding adiponectin quantitation or differences in the studied groups involved.

MMP-1, -3, and -13 are shown to have crucial roles in the pathogenesis of OA. During the course of OA, IL-1 produced by chondrocytes and synovial nucleated cells, activates MMP in cartilage, causing degradation of cartilage matrix [31]. Sachdeva et al. reported that MMP-9 in SF and MMP-13 levels in serum and SF decreased in K-L grade 4 cases [32]. However, there is still need for studies evaluating biochemical biomarkers of different stages of GOA. In this study, a significance was found towards positive correlations between clinical scale scoring and the SF and serum levels of MMP-9.

Adiponectin has been found to enhance nitric oxide, IL-6, MMP-1 and MMP-3 production in OA cartilage [33]. In this study, SF adipokine levels tended to correlate to SF MMP-1, which may confirm the influence of adiponectin on MMP-1 production, probably via IL-1. On the other hand, the synovial MMP-1 levels observed in this study were much higher than the serum MMP-1 levels, which on the contrary maintained the adiponectin levels. Additionally, adiponectin might exert significant pro-inflammatory effects on chondrocytes by inducing the expression of NOS2 and also by the release of strongly stimulating IL-6, MMP-3, MMP-9 and MCP-1 [34]. Sachdeva et al. reported higher levels of leptin and lower levels of adiponectin in SF of patients with advanced OA compared to patients with lower grades of OA. In our study, we were not able to find any correlation of SF and serum adipokine levels to MMP-9 and TIMP-1, which was also reported by other researches [35].

Some research has found a significant correlation between leptin messenger RNA (mRNA) expression in cartilage in patients with advanced OA and BMI, suggesting that leptin could serve as a metabolic link between obesity and OA [36,37,38]. The strong correlation between concentrations of leptin in SF and serum was observed in this study, making leptin a possible OA biomarker in obese females. In this study, lower leptin levels were observed in synovial fluid compared to serum, similar to the results of other authors [39,40] and to rheumatoid arthritis [41]. Previous studies also found that leptin levels were associated with OA patients’ pain [42]. Our study found a positive correlation between both leptin levels, in SF and serum, and BMI, which is consistent with the results of other studies [43,44]. Subcutaneous adipose tissue has been found to be a major determinant of circulating leptin levels [45,46].

In the current study, no significant associations between leptin levels and any of the clinical scores were found. However, there are some studies showing growing SF leptin concentrations in more advanced OA stages [40]. Staikos found increased levels of plasma leptin and decreased levels of SF leptin with increased disease severity [22]. The latter tendency is also observed in this study. The higher baseline serum leptin was associated with greater risk of severe knee joint damage on MRI after a period of 10 years [47]. Additionally, Stannus et al. demonstrated a negative association between serum leptin levels and cartilage volume [48], which shows a possible leptin impact on cartilage degeneration. Leptin mRNA expression in OA cartilage and the levels of leptin and leptin receptor expression in human OA cartilage were related to severity of cartilage destruction [49].

Leptin participates in the regulatory mechanisms of the immune system. It enhances the production of proinflammatory factors and destructive mediators participating in OA pathogenesis, such as MMP-1, MMP-3, MMP-9 and MMP-13 in chondrocytes [44]. In a study by Bao et al., leptin in SF was found to enhance the expression of MMP-2 and MMP-9 [50]. However, in this study, SF and serum levels of leptin did not correlate with levels of MMP-1, MMP-9 and TIMP-1.

The study has some possible limitations; mainly that the control group was not established. In our population during the time of the study, we were not able to collect the group of normal-weight females undergoing the TKA and fulfilling inclusion criteria. Additionally, the sample size was estimated retrospectively and it should be noted that the appropriate power of the test (80%) was not achieved when assessing the correlation between the tested markers assessed in blood or SF and the clinical status of patients. Therefore, the results obtained in the course of analyses should be treated as a preliminary study and further prospective study with increased number of participants is needed to confirm the observed statistical dependencies and tendencies. The use of serum instead of plasma is a weakness of the study, as plasma is the preferred clinical specimen for measurement of several biomarkers (including cytokines, MMPs, etc.). The release of MMPs following degranulation of leukocytes and platelets during blood clotting “blurs” the results (is dependent of platelets and white blood cells numbers).

To conclude, this study confirms the correlations between concentrations of both leptin and ACRP-30, comparing serum and SF. In overweight females, leptin levels increase with BMI and ACRP-30 serum levels increase in more advanced GOA stages. Finally, leptin levels were correlated with TIMP-1 serum concentration, one of the biochemical markers of GOA.

## Figures and Tables

**Table 1:** Demographic and clinical characteristic of the studied group.

Variable	Mean Value ± SD or *n* (%)	Minimum	Maximum
Number of Patients	39		
Age (years)	69.10 ± 6.46	52	85
Body Mass Index (kg/m^2^)	31.52 ± 5.55	25.48	46.90
Duration of symptoms (years)	7.13 ± 6.38	2	40
Knee Society Score (clinical)	29.33 ± 16.19	0	64
Knee Society Score (functional)	41.92 ± 23.30	5	90
Ahlbӓck Score	3.05 ± 1.10	1	5
Kellgren–Lawrence Score	3.69 ± 0.57	2	4

**Table 2:** Concentrations of the studied biomarkers in blood serum and synovial fluid.

Variable	Mean Value ± SD	Min.	Max.
ACRP-30 serum (ng/mL)	8393.80 ± 4093.68	1657.57	17,475.95
ACRP-30 synovial fluid (ng/mL)	774.33 ± 500.54	55.05	2185.97
pro-MMP-1 serum (ng/mL)	0.66 ± 1.00	0.01	6.01
pro-MMP-1 synovial fluid (ng/mL)	17.91 ± 9.99	2.59	33.67
MMP-9 serum (ng/mL)	100.27 ± 84.37	1.10	384.18
MMP-9 synovial fluid (ng/mL)	41.64 ± 56.96	0.10	270.88
TIMP-1 serum (ng/mL)	118.78 ± 54.62	54.024	328.6
TIMP-1 synovial fluid (ng/mL)	428.93 ± 190.49	73.223	869.4
Leptin serum (pg/mL)	32,040.74 ± 28,366.77	3816.667	140,711.1
Leptin synovial fluid (pg/mL)	27,332.12 ± 27,792.92	661.111	118,594.4

**Table 3:** Correlations between the levels of studied biomarkers and clinical status.

VARIABLE	AGE (Years)		BMI		KSS Clinical		KSS Functional		Ahlbäck Score		Kellgren–Lawrence Score	
	Unadjusted	Adjusted	Unadjusted	Adjusted	Unadjusted	Adjusted	Unadjusted	Adjusted	Unadjusted	Adjusted	Unadjusted	Adjusted
ACRP-30 serum (ng/mL)	0.169	0.113	−0.150	−0.045	−0.254	−0.258	0.024	0.200	0.082	0.393	−0.148	−0.415
	0.2962	0.5234	0.3558	0.7999	0.1145	0.1409	0.8850	0.2571	0.6202	0.0214 *	0.3690	0.0146 *
ACRP-30 synovial fluid (ng/mL)	−0.041	−0.212	−0.059	−0.076	0.021	0.062	0.105	−0.040	−0.015	0.197	−0.178	−0.181
	0.8024	0.2291	0.7180	0.6698	0.8998	0.7264	0.5197	0.8235	0.9257	0.2638	0.2783	0.3058
pro-MMP-1 serum(ng/mL)	−0.130	−0.134	0.004	−0.287	0.063	0.218	−0.209	−0.219	−0.126	−0.113	0.045	0.057
	0.4237	0.4497	0.9785	0.0995 ^	0.6986	0.2154	0.1959	0.2142	0.4463	0.5258	0.7873	0.7483
pro-MMP-1 synovial fluid (ng/mL)	0.114	−0.066	−0.156	−0.242	−0.073	−0.038	−0.183	−0.275	0.066	0.058	−0.096	−0.082
	0.4826	0.7089	0.3358	0.1679	0.6522	0.8290	0.2580	0.1151	0.6915	0.7450	0.5597	0.6432
MMP-9 serum (ng/mL)	−0.175	−0.186	0.033	0.487	0.027	0.336	−0.276	−0.361	−0.023	0.279	−0.021	−0.085
	0.2789	0.2913	0.8406	0.0035 *	0.8701	0.0517 ^	0.0849 ^	0.0360 *	0.8902	0.1094	0.8978	0.6320
MMP-9 synovial fluid (ng/mL)	0.205	0.210	−0.109	−0.157	−0.187	−0.093	−0.190	−0.302	0.272	0.203	0.110	−0.178
	0.2048	0.2341	0.5049	0.3749	0.2481	0.5993	0.2402	0.0826 ^	0.0944 ^	0.2490	0.5039	0.3135
TIMP-1 serum (ng/mL)	0.132	0.235	−0.040	0.1828	0.101	0.079	−0.057	0.134	−0.134	−0.043	−0.011	0.091
	0.4229	0.1804	0.8076	0.3006	0.5396	0.6566	0.7281	0.4508	0.4162	0.8094	0.9488	0.6077
TIMP-1 synovial fluid (ng/mL)	0.209	0.218	−0.178	0.038	−0.260	−0.093	−0.212	−0.187	0.297	0.179	0.173	−0.034
	0.2017	0.2150	0.2793	0.8305	0.1094	0.6021	0.1956	0.2894	0.0666 ^	0.3123	0.2908	0.8500
Leptin serum (pg/mL)	−0.030	0.082	0.553	0.568	0.159	0.179	−0.016	0.030	−0.221	0.045	−0.235	−0.126
	0.8532	0.6448	0.0003	0.0005 *	0.3262	0.3120	0.9229	0.8662	0.1698	0.8018	0.1448	0.4776
Leptin synovial fluid (pg/mL)	−0.047	−0.013	0.549	0.590	0.113	0.175	0.025	−0.016	−0.201	0.187	−0.258	−0.192
	0.7726	0.9398	0.0003	0.0002 *	0.4860	0.3224	0.8786	0.9287	0.2137	0.2894	0.1075	0.2773

* statistically significant result, ^ a tendency for a statistically significant result. Results were adjusted by age, BMI, KSS (clinical and functional), Ahlbäck score and Kellgren–Lawrence score.

**Table 4:** Correlations between the levels of studied biomarkers.

Variable	ACRP-30 Serum (ng/mL)	ACRP-30 Synovial Fluid (ng/mL)	Pro-MMP-1 Serum (ng/mL)	Pro-MMP-1 Synovial Fluid (ng/mL)	MMP-9 Serum (ng/mL)	MMP-9 Synoviafluid (ng/mL)	TIMP-1 Serum (ng/mL)	TIMP-1 Synovialfluid (ng/mL)	Leptin Serum (pg/mL)	Leptin Synovial Fluid (pg/mL)
	R-Spearman p									
ACRP-30 synovial fluid (ng/mL)	0.541	-	-	-	-	-	-	-	-	-
	0.0003 *									
pro-MMP-1 serum (ng/mL)	−0.092	−0.300	-	-	-	-	-	-	-	-
	0.5734	0.0598 ^								
pro-MMP-1 synovial fluid (ng/mL)	0.090	0.300	−0.051	-	-	-	-	-	-	-
	0.5797	0.0600 ^	0.7527							
MMP-9 serum (ng/mL)	−0.035	−0.056	0.411	0.077	-	-	-	-	-	-
	0.8316	0.7297	0.0085 *	0.6379						
MMP-9 synovial fluid (ng/mL)	0.130	0.004	0.030	0.380	0.014	-	-	-	-	-
	0.4242	0.9798	0.8559	0.0155 *	0.9336					
TIMP-1 serum (ng/mL)	0.015	−0.183	0.264	−0.039	0.257	−0.160	-	-	-	-
	0.9298	0.2654	0.1041	0.8133	0.1148	0.3301				
TIMP-1 synovial fulid (ng/mL)	0.099	0.002	−0.176	0.012	0.266	0.016	0.090	-	-	-
	0.5480	0.9902	0.2841	0.9444	0.1017	0.9216	0.5864			
Leptin serum (pg/mL)	0.122	−0.028	0.219	0.079	−0.002	−0.079	0.442	0.186	-	-
	0.4646	0.8667	0.1859	0.6365	0.9886	0.6388	0.0054 *	0.2631		
Leptin synovial fluid (pg/mL)	0.204	0.180	0.222	0.061	−0.100	−0.180	0.267	0.116	0.778	-
	0.2191	0.2798	0.1805	0.7175	0.5487	0.2789	0.1057	0.4894	<0.0001 *	

* statistically significant result, ^ a tendency for a statistically significant result.

## Data Availability

The data are available from corresponding author on demand.

## Supplementary Materials

The following are available online at https://www.mdpi.com/2077-0383/10/6/1263/s1, Figure S1: Box plots (Box and Whiskers) of studied markers (concentration measurements in serum and synovial fluid). A. ACRP-30. B. pro-MMP1. C. MMP9. D. TIMP1. E. Leptin.
