# Supplementary material for: Level of Adiponectin, Leptin and Selected Matrix Metalloproteinases in Female Overweight Patients with Primary Gonarthrosis
Source: J Clin Med. 2021 Mar 18;10(6):1263. doi: 10.3390/jcm10061263 (PMC8003316; doi:10.3390/jcm10061263)
Supplement: Supplementary file 1 [file jcm-10-01263-s001.pdf]

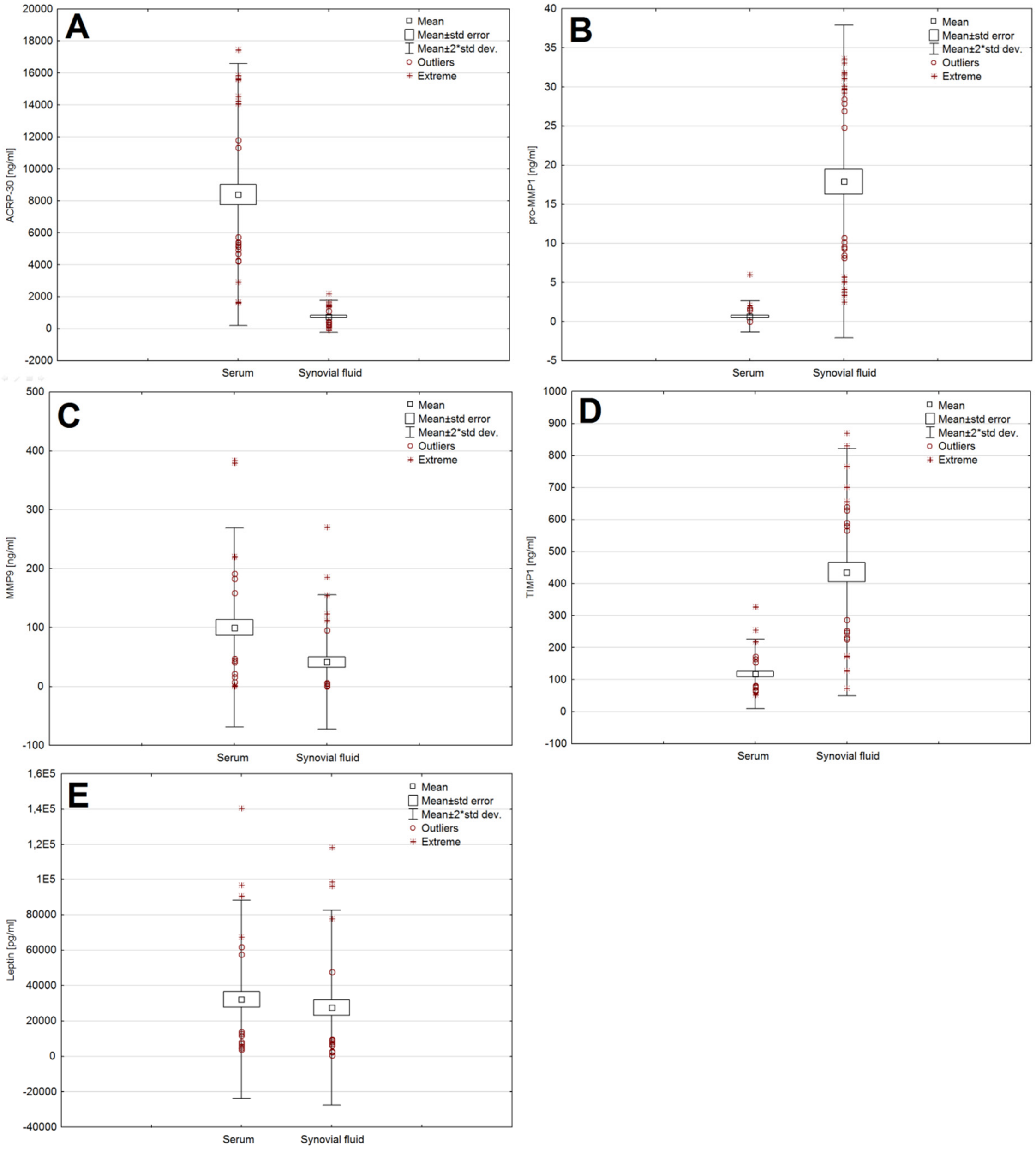

Figure S1. Box plots (Box and Whiskers) of studied markers (concentration measurements in serum and synovial fluid). A. ACRP-30. B. pro-MMP1. C. MMP9. D. TIMP1. E. Leptin.
